# Supplementary figures and images for: Maternal obesity and metabolic disorders associate with congenital heart defects in the offspring: A systematic review
Source: PLoS One. 2021 May 27;16(5):e0252343. doi: 10.1371/journal.pone.0252343 (PMC8158948; doi:10.1371/journal.pone.0252343)

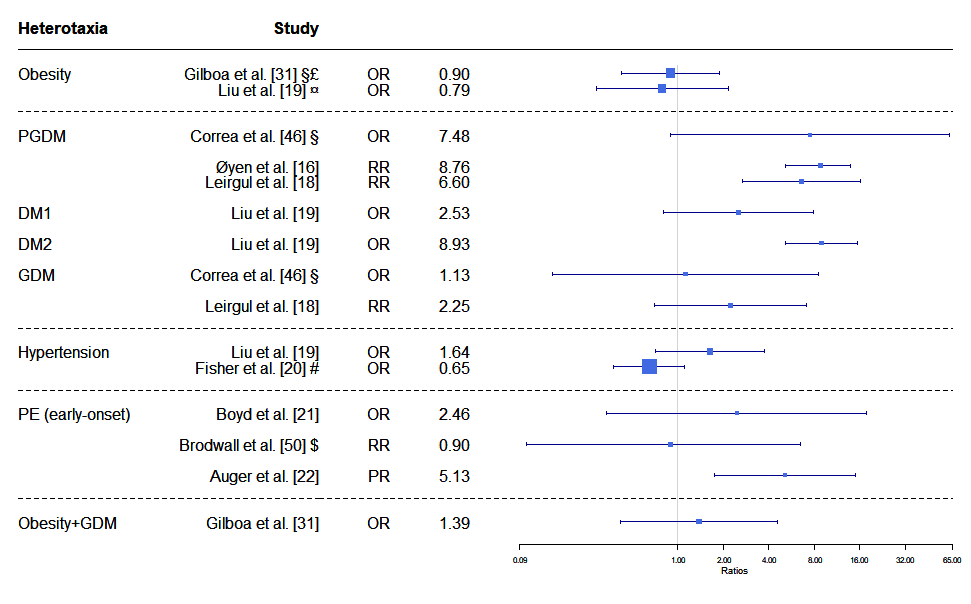

Supplement: S1 Fig — Obesity is defined as BMI ≥ 30 kg/m2 unless other is stated; early-onset PE defined as debut before gestational week 34; PGDM are defined as DM1 or DM2; all risk estimates are adjusted unless other is stated; *, not adjusted; §, isolated defects; ¤, obesity defined from ICD-10 codes; £, BMI ≥ 35 kg/m2; €, BMI > 29 kg/m2; †, BMI 35-<40 kg/m2; #, untreated hypertension; $, Brodwall et al. pooled early-onset PE and severe PE. Abbreviations: BMI, body mass index; DM1, diabetes type 1; DM2, diabetes type 2; GDM, gestational diabetes; OR, odds ratio; PE, preeclampsia; PGDM, pregestational diabetes; PR, prevalence ratio; RR, risk ratio. (TIF) [file pone.0252343.s001.tif]

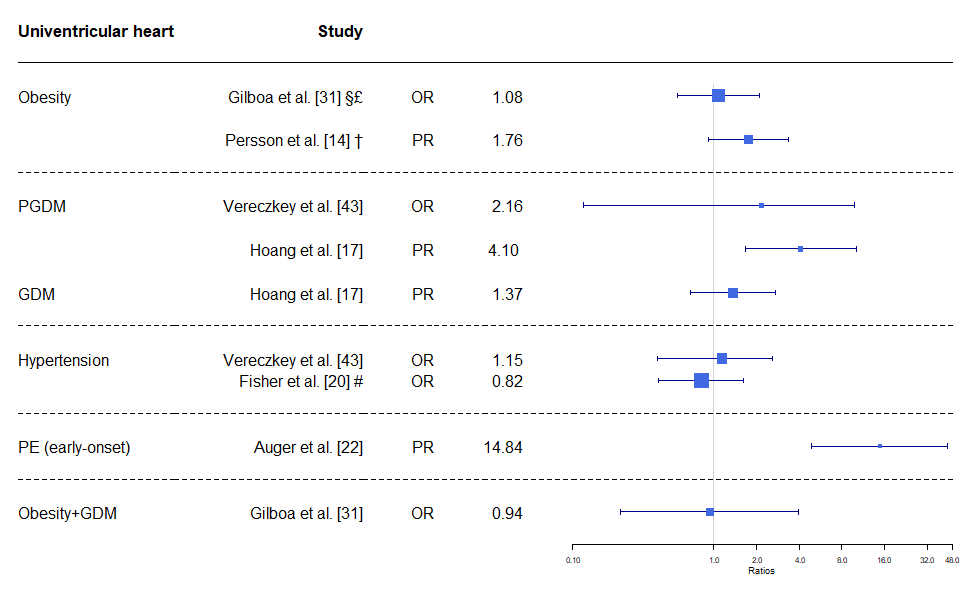

Supplement: S2 Fig — Obesity is defined as BMI ≥ 30 kg/m2 unless other is stated; early-onset PE defined as debut before gestational week 34; PGDM are defined as DM1 or DM2; all risk estimates are adjusted unless other is stated; *, not adjusted; £, BMI ≥ 35 kg/m2; †, BMI 35-<40 kg/m2; #, untreated hypertension. Abbreviations: BMI, body mass index; DM1, diabetes type 1; DM2, diabetes type 2; GDM, gestational diabetes; OR, odds ratio; PE, preeclampsia; PGDM, pregestational diabetes; PR, prevalence ratio; RR, risk ratio. (TIF) [file pone.0252343.s002.tif]

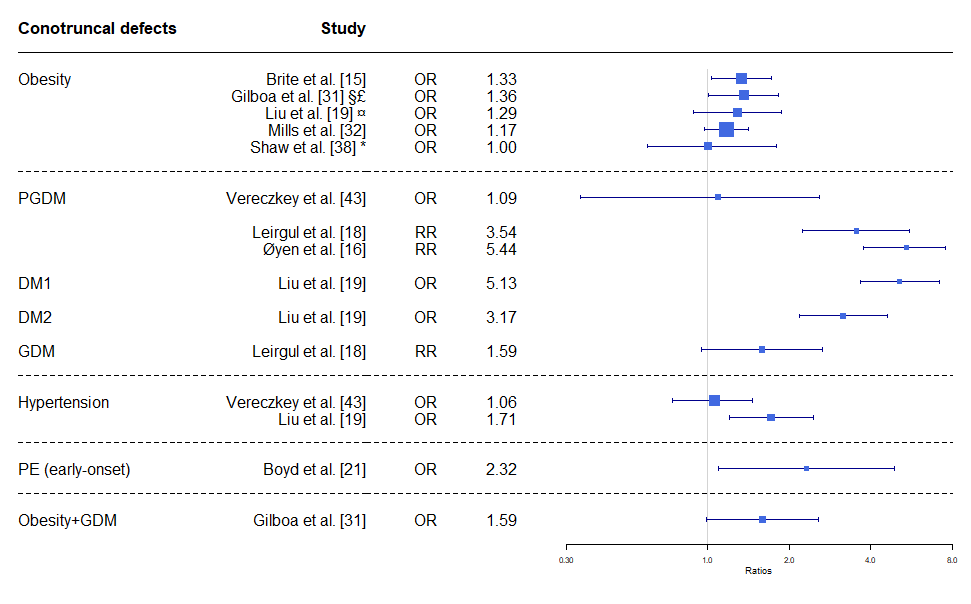

Supplement: S3 Fig — Obesity is defined as BMI ≥ 30 kg/m2 unless other is stated; early-onset PE defined as debut before gestational week 34; PGDM are defined as DM1 or DM2; all risk estimates are adjusted unless other is stated; *, not adjusted; §, isolated defects; ¤, obesity defined from ICD-10 codes; £, BMI ≥ 35 kg/m2. Abbreviations: BMI, body mass index; DM1, diabetes type 1; DM2, diabetes type 2; GDM, gestational diabetes; OR, odds ratio; PE, preeclampsia; PGDM, pregestational diabetes; PR, prevalence ratio; RR, risk ratio. (TIF) [file pone.0252343.s003.tif]

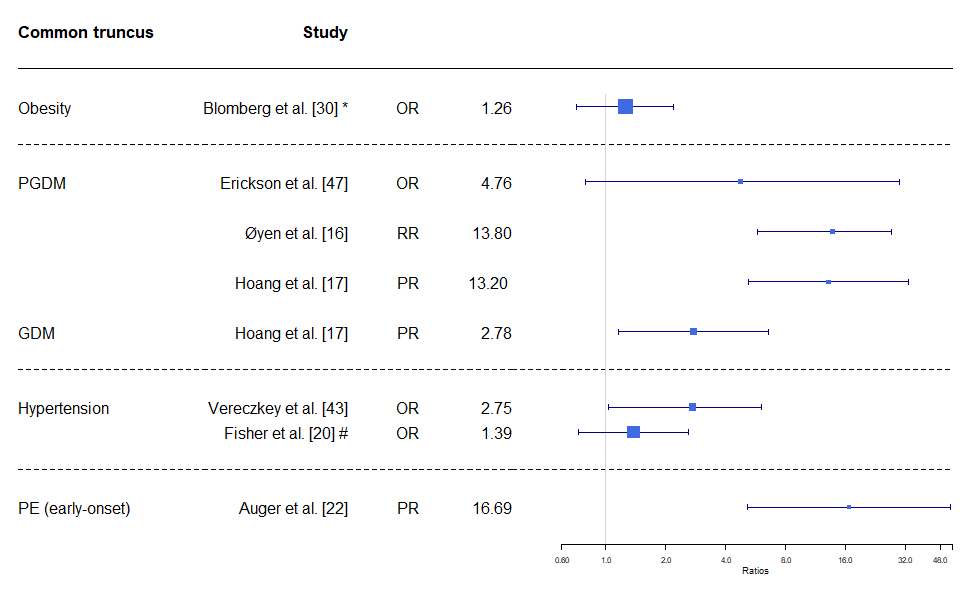

Supplement: S4 Fig — Obesity is defined as BMI ≥ 30 kg/m2 unless other is stated; early-onset PE defined as debut before gestational week 34; PGDM are defined as DM1 or DM2; all risk estimates are adjusted unless other is stated; *, not adjusted; #, untreated hypertension. Abbreviations: BMI, body mass index; DM1, diabetes type 1; DM2, diabetes type 2; GDM, gestational diabetes; OR, odds ratio; PE, preeclampsia; PGDM, pregestational diabetes; PR, prevalence ratio; RR, risk ratio. (TIF) [file pone.0252343.s004.tif]

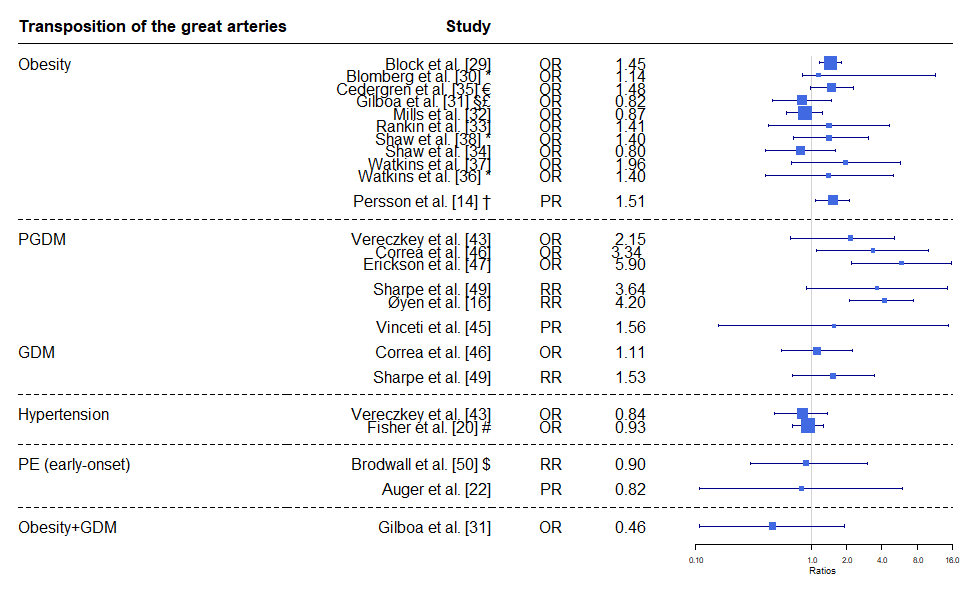

Supplement: S5 Fig — Obesity is defined as BMI ≥ 30 kg/m2 unless other is stated; early-onset PE defined as debut before gestational week 34; PGDM are defined as DM1 or DM2; all risk estimates are adjusted unless other is stated; *, not adjusted; §, isolated defects; £, BMI ≥ 35 kg/m2; €, BMI > 29 kg/m2; †, BMI 35-<40 kg/m2; #, untreated hypertension; $, Brodwall et al. pooled early-onset PE and severe PE. Abbreviations: BMI, body mass index; DM1, diabetes type 1; DM2, diabetes type 2; GDM, gestational diabetes; OR, odds ratio; PE, preeclampsia; PGDM, pregestational diabetes; PR, prevalence ratio; RR, risk ratio. (TIF) [file pone.0252343.s005.tif]

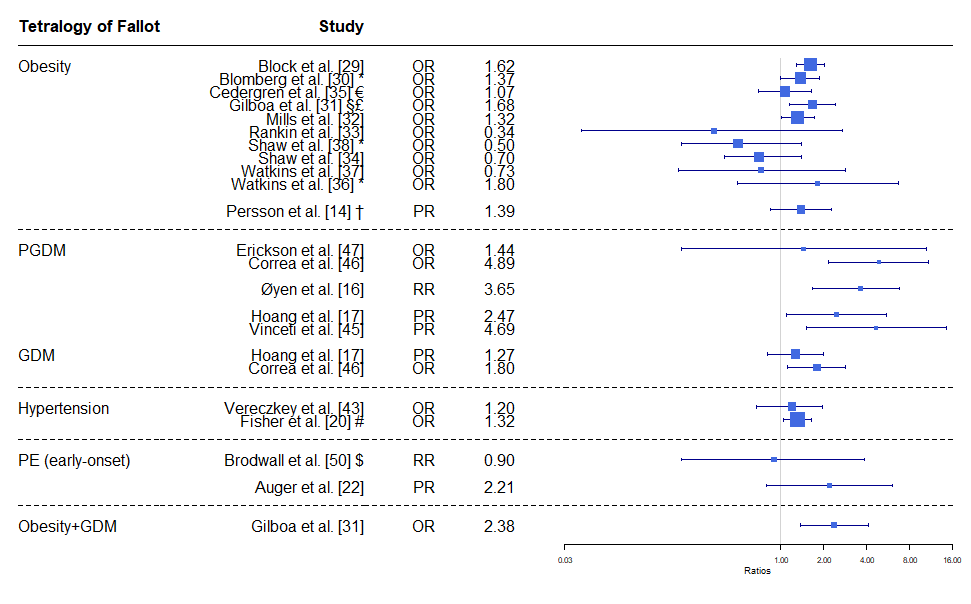

Supplement: S6 Fig — Obesity is defined as BMI ≥ 30 kg/m2 unless other is stated; early-onset PE defined as debut before gestational week 34; PGDM are defined as DM1 or DM2; all risk estimates are adjusted unless other is stated; *, not adjusted; §, isolated defects; £, BMI ≥ 35 kg/m2; €, BMI > 29 kg/m2; †, BMI 35-<40 kg/m2; #, untreated hypertension; $, Brodwall et al. pooled early-onset PE and severe PE. Abbreviations: BMI, body mass index; DM1, diabetes type 1; DM2, diabetes type 2; GDM, gestational diabetes; OR, odds ratio; PE, preeclampsia; PGDM, pregestational diabetes; PR, prevalence ratio; RR, risk ratio. (TIF) [file pone.0252343.s006.tif]

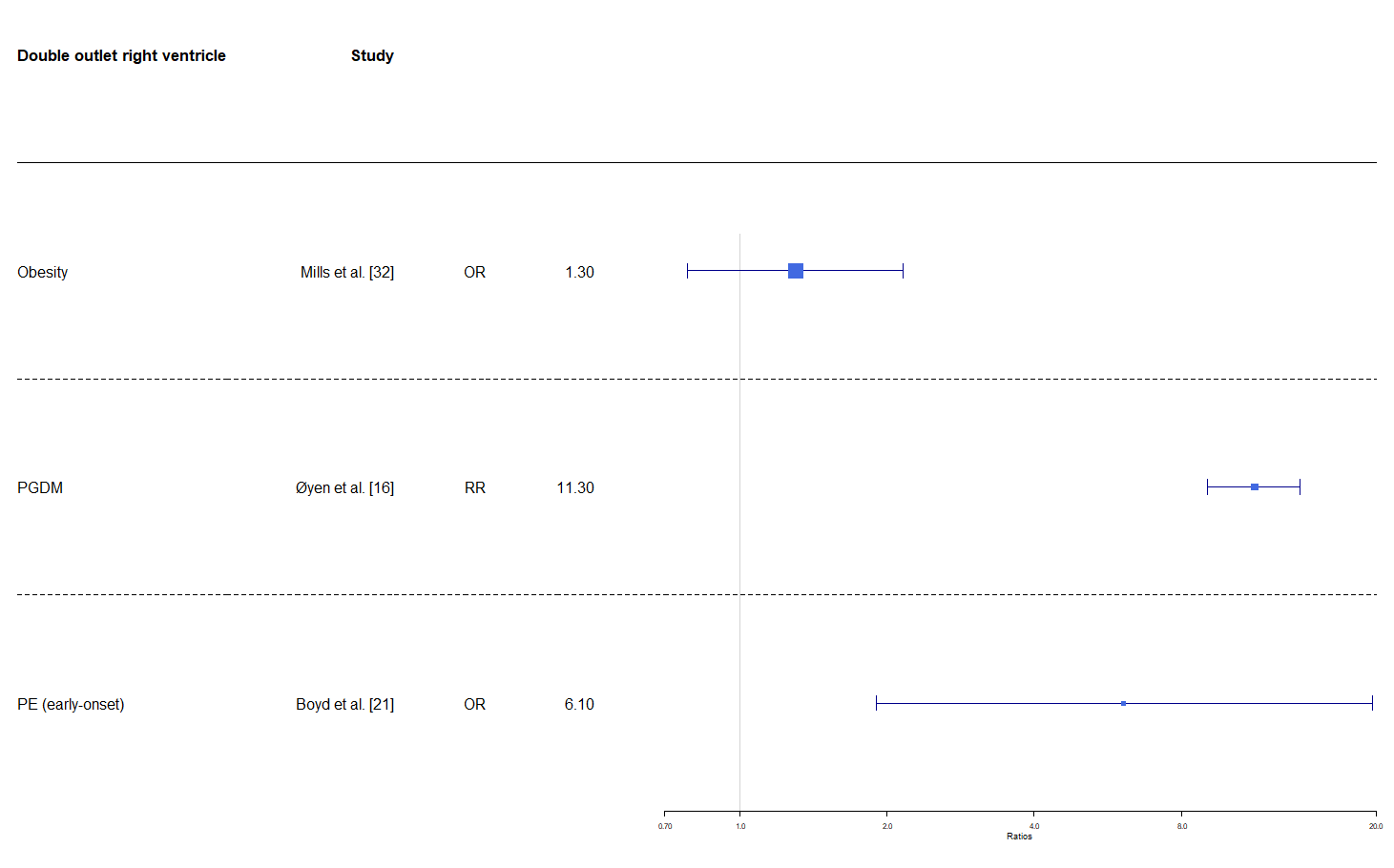

Supplement: S7 Fig — Obesity is defined as BMI ≥ 30 kg/m2 unless other is stated; early-onset PE defined as debut before gestational week 34; PGDM are defined as DM1 or DM2; all risk estimates are adjusted unless other is stated; *, not adjusted. Abbreviations: BMI, body mass index; DM1, diabetes type 1; DM2, diabetes type 2; GDM, gestational diabetes; OR, odds ratio; PE, preeclampsia; PGDM, pregestational diabetes; PR, prevalence ratio; RR, risk ratio. (TIF) [file pone.0252343.s007.tif]

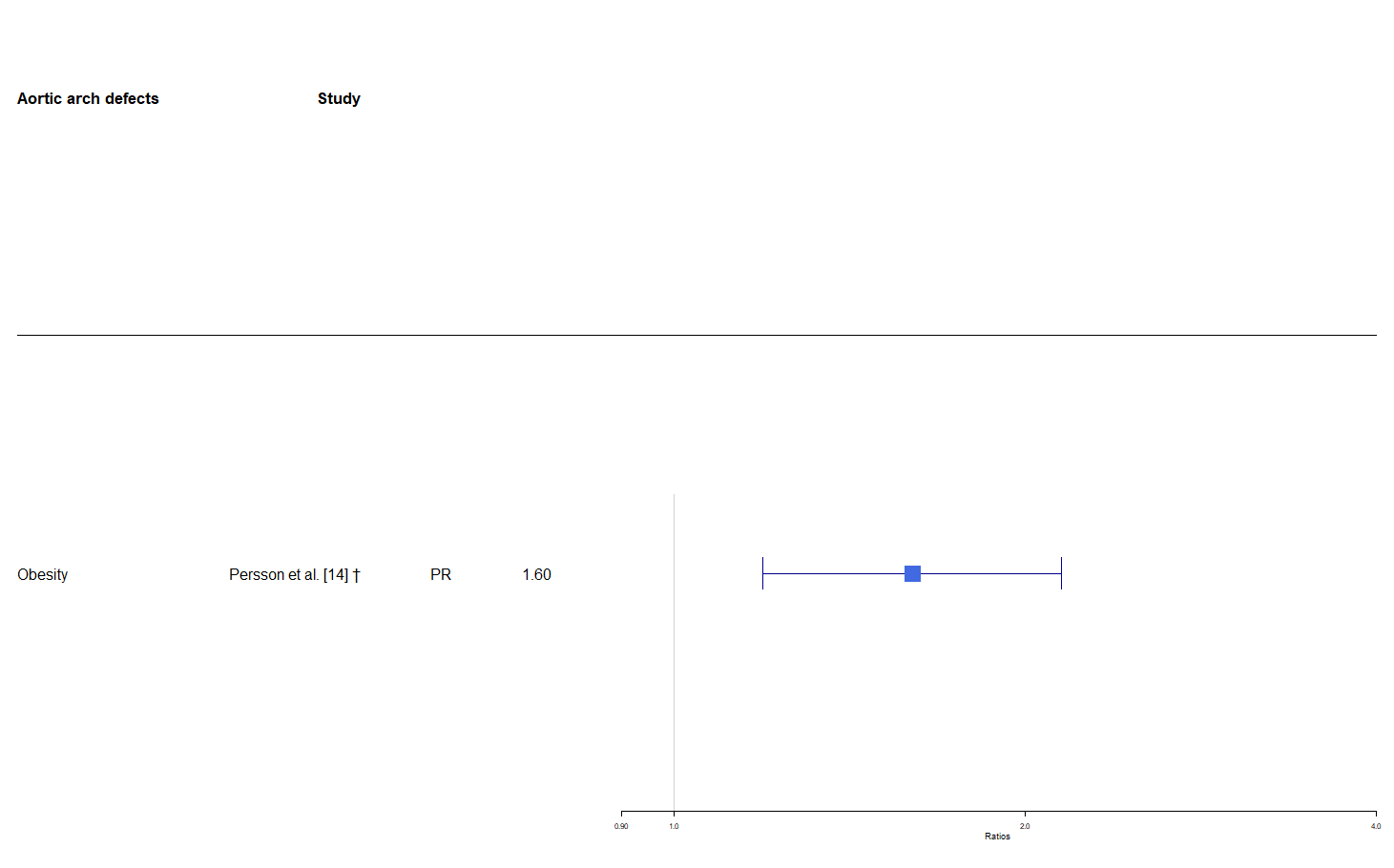

Supplement: S8 Fig — Obesity is defined as BMI ≥ 30 kg/m2 unless other is stated; early-onset PE defined as debut before gestational week 34; PGDM are defined as DM1 or DM2; all risk estimates are adjusted unless other is stated; *, not adjusted; †, BMI 35-<40 kg/m2. Abbreviations: BMI, body mass index; DM1, diabetes type 1; DM2, diabetes type 2; GDM, gestational diabetes; OR, odds ratio; PE, preeclampsia; PGDM, pregestational diabetes; PR, prevalence ratio; RR, risk ratio. (TIF) [file pone.0252343.s008.tif]

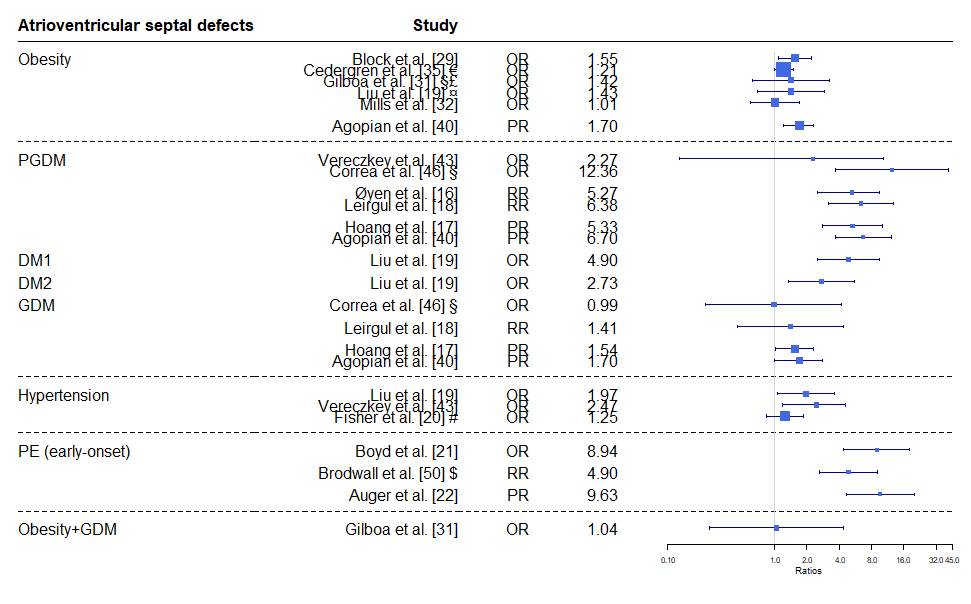

Supplement: S9 Fig — Obesity is defined as BMI ≥ 30 kg/m2 unless other is stated; early-onset PE defined as debut before gestational week 34; PGDM are defined as DM1 or DM2; all risk estimates are adjusted unless other is stated; *, not adjusted; §, isolated defects; ¤, obesity defined from ICD-10 codes; £, BMI ≥ 35 kg/m2; €, BMI > 29 kg/m2; #, untreated hypertension; $, Brodwall et al. pooled early-onset PE and severe PE. Abbreviations: BMI, body mass index; DM1, diabetes type 1; DM2, diabetes type 2; GDM, gestational diabetes; OR, odds ratio; PE, preeclampsia; PGDM, pregestational diabetes; PR, prevalence ratio; RR, risk ratio. (TIF) [file pone.0252343.s009.tif]

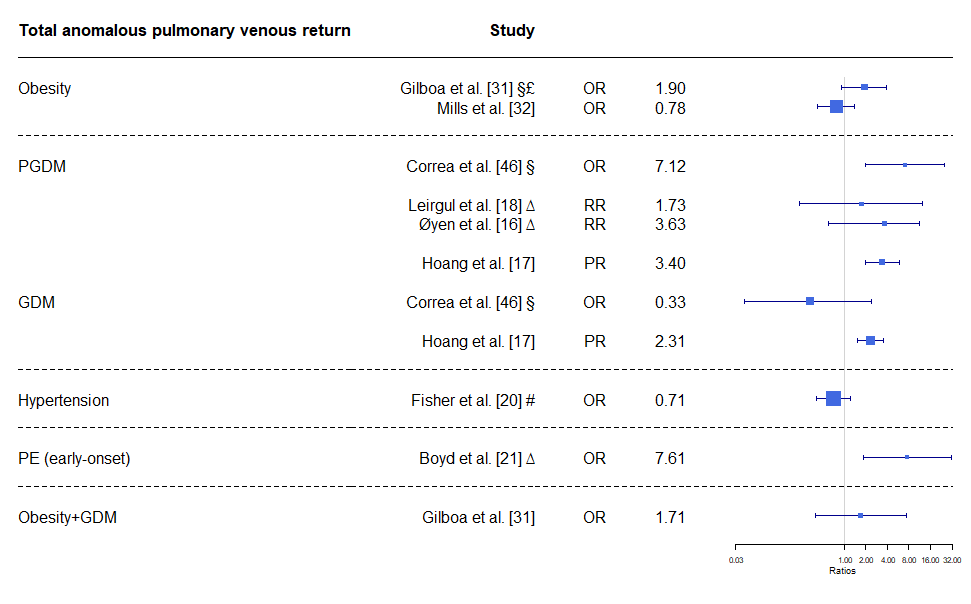

Supplement: S10 Fig — Obesity is defined as BMI ≥ 30 kg/m2 unless other is stated; early-onset PE defined as debut before gestational week 34; PGDM are defined as DM1 or DM2; all risk estimates are adjusted unless other is stated; *, not adjusted; §, isolated defects; £, BMI ≥ 35 kg/m2; Δ, estimates for anomalous pulmonary venous return; #, untreated hypertension. Abbreviations: BMI, body mass index; DM1, diabetes type 1; DM2, diabetes type 2; GDM, gestational diabetes; OR, odds ratio; PE, preeclampsia; PGDM, pregestational diabetes; PR, prevalence ratio; RR, risk ratio. (TIF) [file pone.0252343.s010.tif]

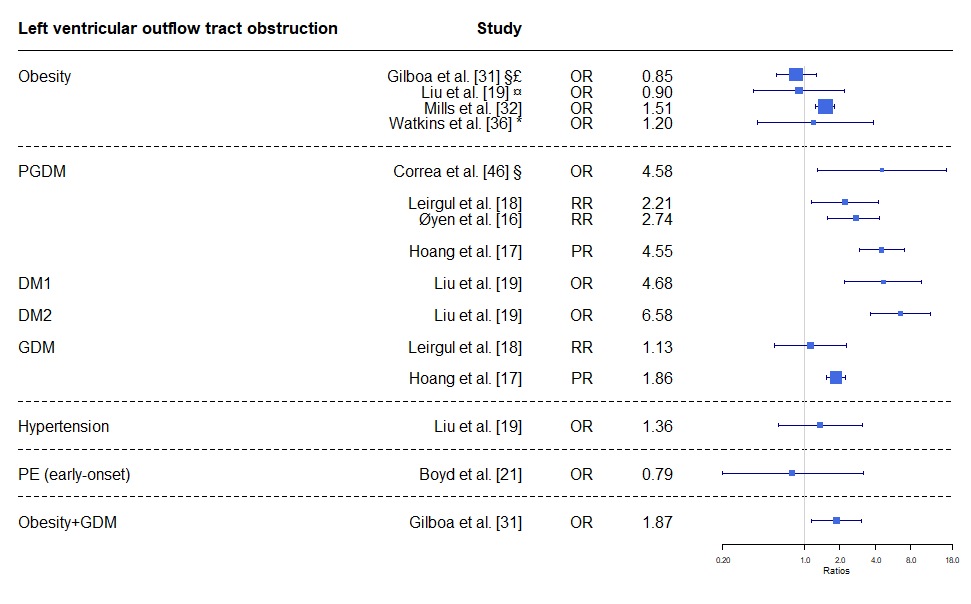

Supplement: S11 Fig — Obesity is defined as BMI ≥ 30 kg/m2 unless other is stated; early-onset PE defined as debut before gestational week 34; PGDM are defined as DM1 or DM2; all risk estimates are adjusted unless other is stated; *, not adjusted; §, isolated defects; ¤, obesity defined from ICD-10 codes; £, BMI ≥ 35 kg/m2. Abbreviations: BMI, body mass index; DM1, diabetes type 1; DM2, diabetes type 2; GDM, gestational diabetes; OR, odds ratio; PE, preeclampsia; PGDM, pregestational diabetes; PR, prevalence ratio; RR, risk ratio. (TIF) [file pone.0252343.s011.tif]

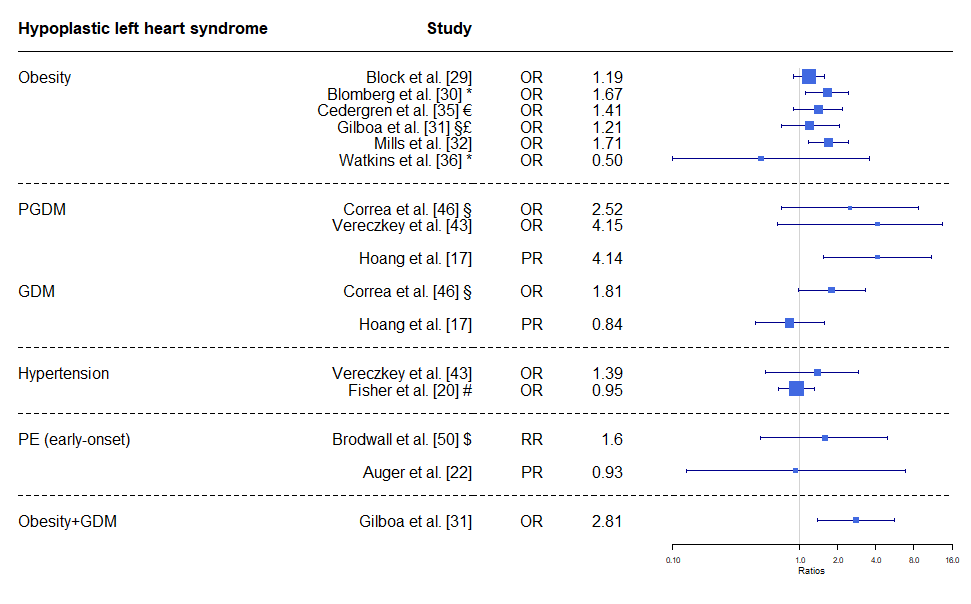

Supplement: S12 Fig — Obesity is defined as BMI ≥ 30 kg/m2 unless other is stated; early-onset PE defined as debut before gestational week 34; PGDM are defined as DM1 or DM2; all risk estimates are adjusted unless other is stated; *, not adjusted; §, isolated defects; £, BMI ≥ 35 kg/m2; €, BMI > 29 kg/m2; #, untreated hypertension; $, Brodwall et al. pooled early-onset PE and severe PE. Abbreviations: BMI, body mass index; DM1, diabetes type 1; DM2, diabetes type 2; GDM, gestational diabetes; OR, odds ratio; PE, preeclampsia; PGDM, pregestational diabetes; PR, prevalence ratio; RR, risk ratio. (TIF) [file pone.0252343.s012.tif]

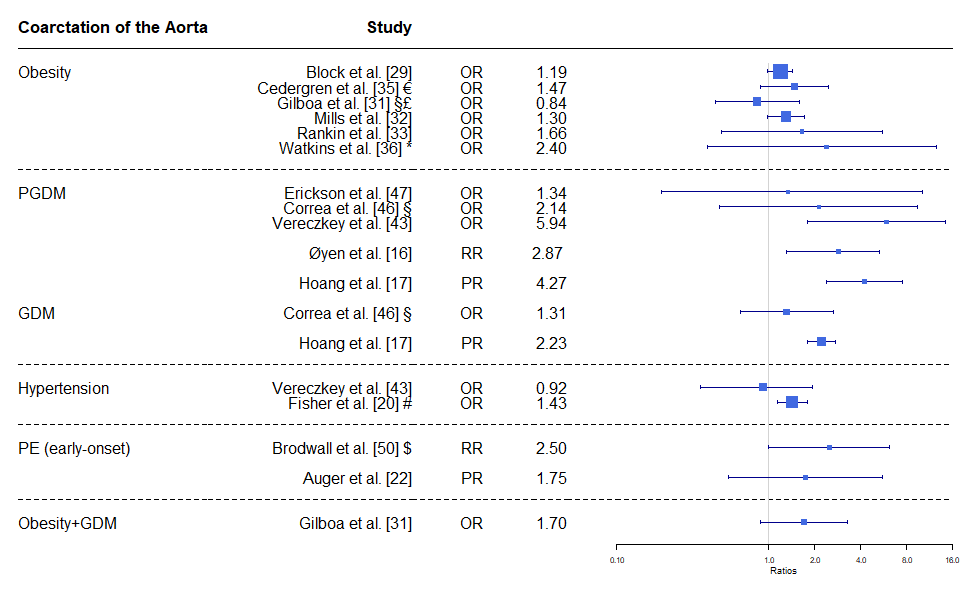

Supplement: S13 Fig — Obesity is defined as BMI ≥ 30 kg/m2 unless other is stated; early-onset PE defined as debut before gestational week 34; PGDM are defined as DM1 or DM2; all risk estimates are adjusted unless other is stated; *, not adjusted; §, isolated defects; £, BMI ≥ 35 kg/m2; €, BMI > 29 kg/m2; #, untreated hypertension; $, Brodwall et al. pooled early-onset PE and severe PE. Abbreviations: BMI, body mass index; DM1, diabetes type 1; DM2, diabetes type 2; GDM, gestational diabetes; OR, odds ratio; PE, preeclampsia; PGDM, pregestational diabetes; PR, prevalence ratio; RR, risk ratio. (TIF) [file pone.0252343.s013.tif]

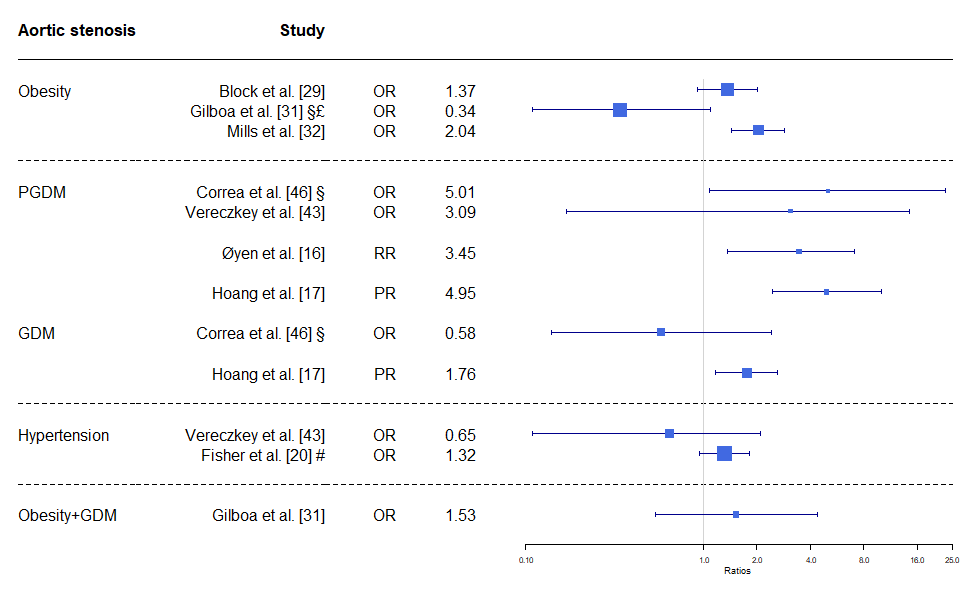

Supplement: S14 Fig — Obesity is defined as BMI ≥ 30 kg/m2 unless other is stated; early-onset PE defined as debut before gestational week 34; PGDM are defined as DM1 or DM2; all risk estimates are adjusted unless other is stated; *, not adjusted; §, isolated defects; £, BMI ≥ 35 kg/m2; #, untreated hypertension. Abbreviations: BMI, body mass index; DM1, diabetes type 1; DM2, diabetes type 2; GDM, gestational diabetes; OR, odds ratio; PE, preeclampsia; PGDM, pregestational diabetes; PR, prevalence ratio; RR, risk ratio. (TIF) [file pone.0252343.s014.tif]

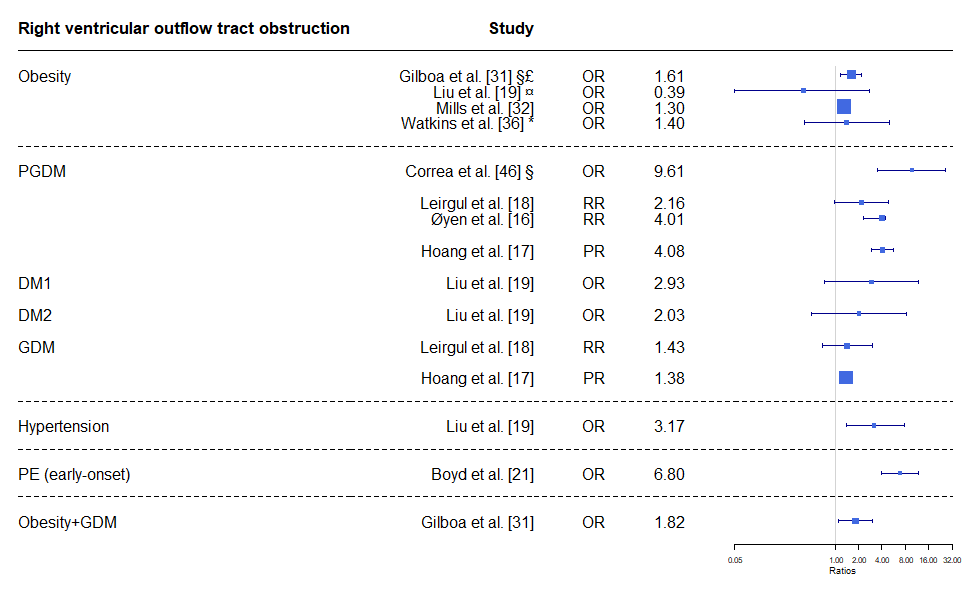

Supplement: S15 Fig — Obesity is defined as BMI ≥ 30 kg/m2 unless other is stated; early-onset PE defined as debut before gestational week 34; PGDM are defined as DM1 or DM2; all risk estimates are adjusted unless other is stated; *, not adjusted; §, isolated defects; £, BMI ≥ 35 kg/m2; #, untreated hypertension. Abbreviations: BMI, body mass index; DM1, diabetes type 1; DM2, diabetes type 2; GDM, gestational diabetes; OR, odds ratio; PE, preeclampsia; PGDM, pregestational diabetes; PR, prevalence ratio; RR, risk ratio. (TIF) [file pone.0252343.s015.tif]

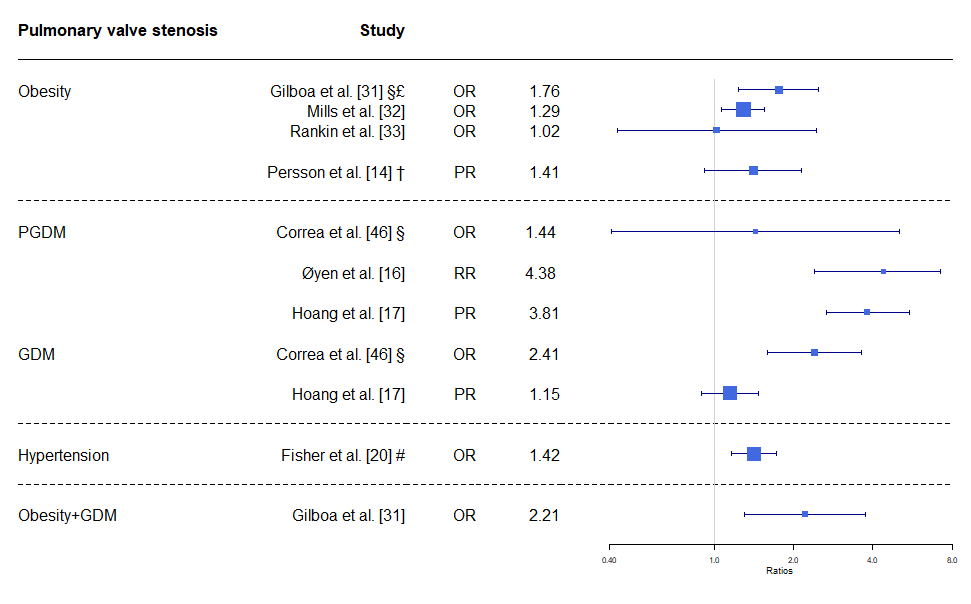

Supplement: S16 Fig — Obesity is defined as BMI ≥ 30 kg/m2 unless other is stated; early-onset PE defined as debut before gestational week 34; PGDM are defined as DM1 or DM2; all risk estimates are adjusted unless other is stated; *, not adjusted; §, isolated defects; £, BMI ≥ 35 kg/m2; †, BMI 35-<40 kg/m2; #, untreated hypertension. Abbreviations: BMI, body mass index; DM1, diabetes type 1; DM2, diabetes type 2; GDM, gestational diabetes; OR, odds ratio; PE, preeclampsia; PGDM, pregestational diabetes; PR, prevalence ratio; RR, risk ratio. (TIF) [file pone.0252343.s016.tif]

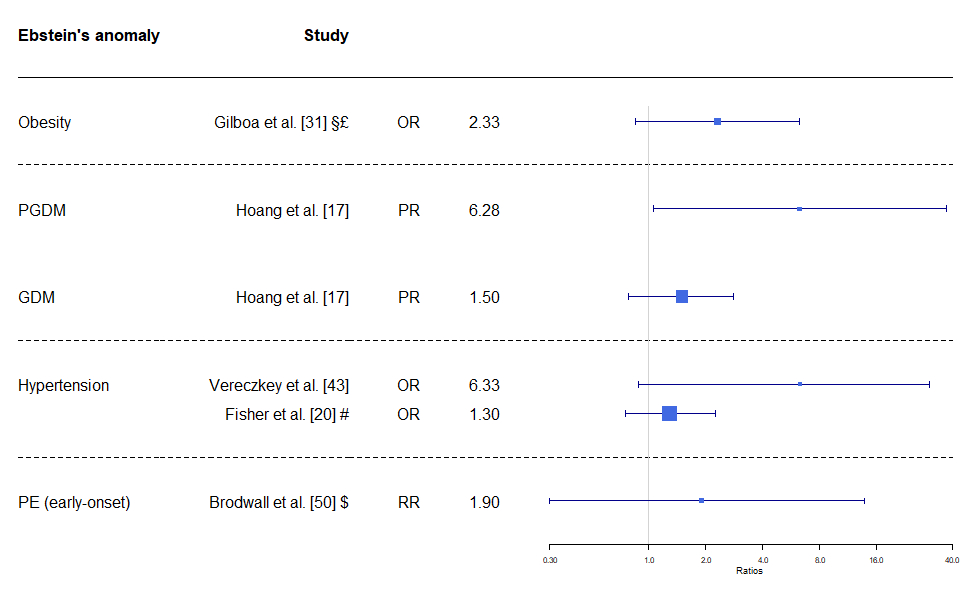

Supplement: S17 Fig — Obesity is defined as BMI ≥ 30 kg/m2 unless other is stated; early-onset PE defined as debut before gestational week 34; PGDM are defined as DM1 or DM2; all risk estimates are adjusted unless other is stated; *, not adjusted; §, isolated defects; £, BMI ≥ 35 kg/m2; #, untreated hypertension; $, Brodwall et al. pooled early-onset PE and severe PE. Abbreviations: BMI, body mass index; DM1, diabetes type 1; DM2, diabetes type 2; GDM, gestational diabetes; OR, odds ratio; PE, preeclampsia; PGDM, pregestational diabetes; PR, prevalence ratio; RR, risk ratio. (TIF) [file pone.0252343.s017.tif]

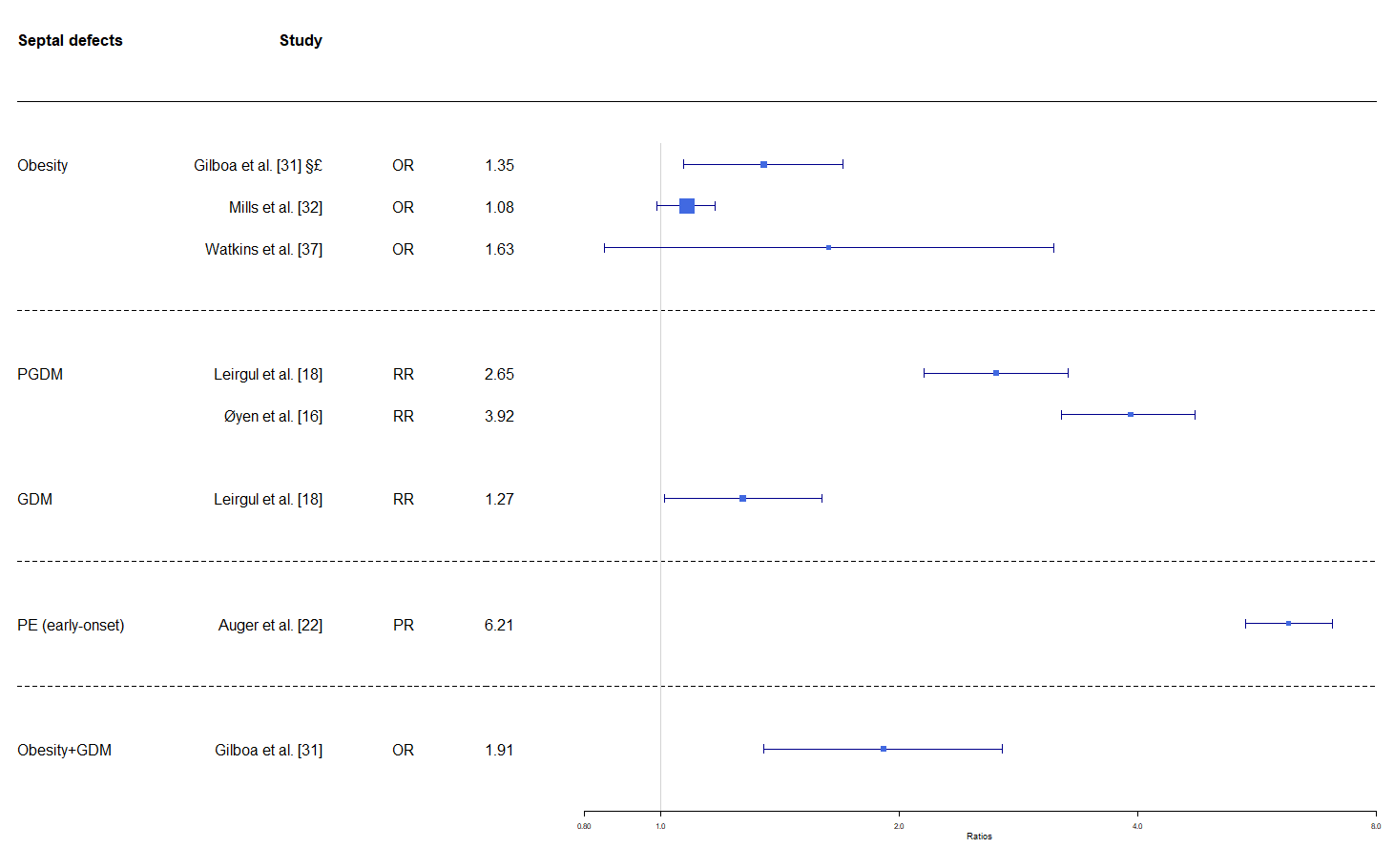

Supplement: S18 Fig — Obesity is defined as BMI ≥ 30 kg/m2 unless other is stated; early-onset PE defined as debut before gestational week 34; PGDM are defined as DM1 or DM2; all risk estimates are adjusted unless other is stated; *, not adjusted; §, isolated defects; £, BMI ≥ 35 kg/m2. Abbreviations: BMI, body mass index; DM1, diabetes type 1; DM2, diabetes type 2; GDM, gestational diabetes; OR, odds ratio; PE, preeclampsia; PGDM, pregestational diabetes; PR, prevalence ratio; RR, risk ratio. (TIF) [file pone.0252343.s018.tif]

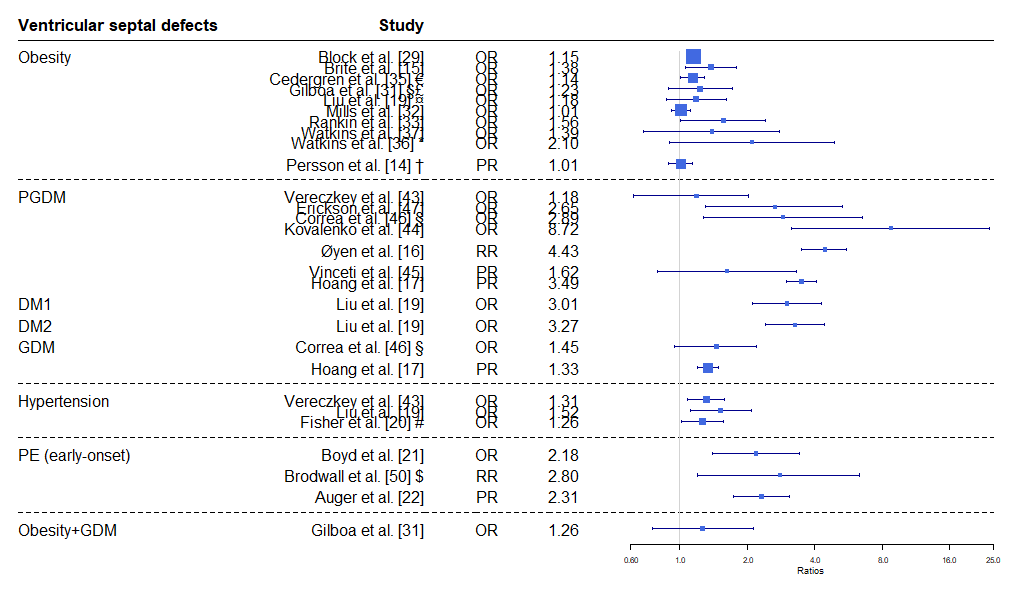

Supplement: S19 Fig — Obesity is defined as BMI ≥ 30 kg/m2 unless other is stated; early-onset PE defined as debut before gestational week 34; PGDM are defined as DM1 or DM2; all risk estimates are adjusted unless other is stated; *, not adjusted; §, isolated defects; ¤, obesity defined from ICD-10 codes; £, BMI ≥ 35 kg/m2; €, BMI > 29 kg/m2; †, BMI 35-<40 kg/m2; #, untreated hypertension; $, Brodwall et al. pooled early-onset PE and severe PE. Abbreviations: BMI, body mass index; DM1, diabetes type 1; DM2, diabetes type 2; GDM, gestational diabetes; OR, odds ratio; PE, preeclampsia; PGDM, pregestational diabetes; PR, prevalence ratio; RR, risk ratio. (TIF) [file pone.0252343.s019.tif]

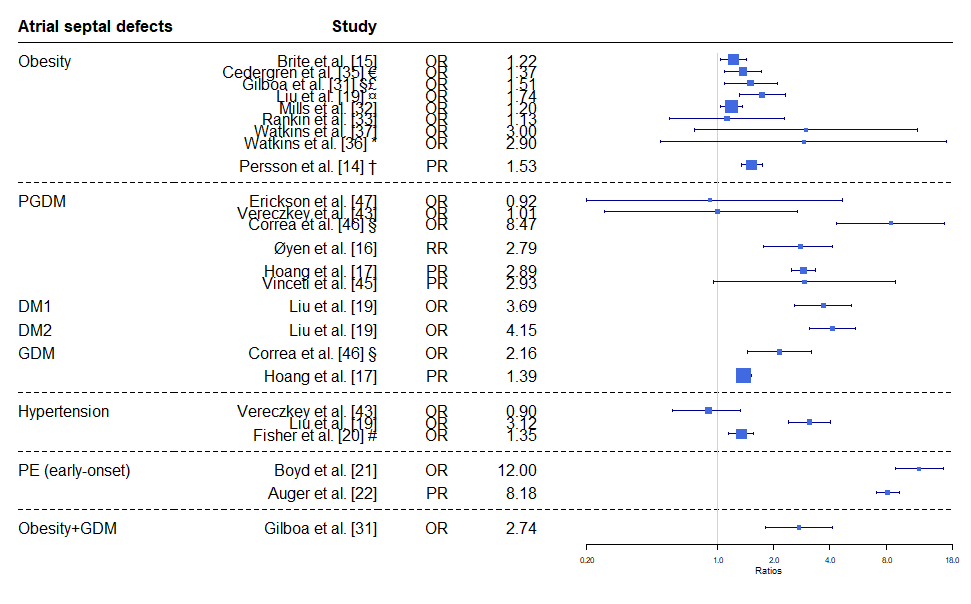

Supplement: S20 Fig — Obesity is defined as BMI ≥ 30 kg/m2 unless other is stated; early-onset PE defined as debut before gestational week 34; PGDM are defined as DM1 or DM2; all risk estimates are adjusted unless other is stated; *, not adjusted; §, isolated defects; ¤, obesity defined from ICD-10 codes; £, BMI ≥ 35 kg/m2; €, BMI > 29 kg/m2; †, BMI 35-<40 kg/m2; #, untreated hypertension. Abbreviations: BMI, body mass index; DM1, diabetes type 1; DM2, diabetes type 2; GDM, gestational diabetes; OR, odds ratio; PE, preeclampsia; PGDM, pregestational diabetes; PR, prevalence ratio; RR, risk ratio. (TIF) [file pone.0252343.s020.tif]
