# Supplementary material for: Maternal obesity and metabolic disorders associate with congenital heart defects in the offspring: A systematic review
Source: PLoS One. 2021 May 27;16(5):e0252343. doi: 10.1371/journal.pone.0252343 (PMC8158948; doi:10.1371/journal.pone.0252343)
Supplement: S1 Table — PubMed and Embase: Publications between January 1, 1990 and October 6, 2019. (PDF) [file pone.0252343.s021.pdf]

Table S1: Search protocol

PubMed and Embase: Publications between January 1, 1990 and January 14, 2021.

Maternal overweight or obesity or metabolic syndrome and CHDs in offspring

PubMed Search Strategy

	Aspect 1	Aspect 2	Aspect 3
MeSH	Heart defects, congenital	Body Mass Index OR Overweight OR Adiposity OR Thinness OR Metabolic Syndrome	Pregnancy OR Infant OR Fetus
Free text search	Congenital heart defect* OR Congenital heart malformation* OR CHD OR Congenital heart disease* OR “Cardiac abnormalities” OR “Cardiac abnormality” OR Cardiac defect* OR Fetal heart OR Fetal cardiac defect* OR Foetal heart OR Foetal cardiac defect*	BMI OR Maternal overweight OR Maternal obesity OR Prepregnancy BMI OR Prepregnancy weight	Offspring* OR Pregnancy

PubMed Search 2019-10-06

		No. of articles
#1	"Heart Defects, Congenital"[Majr]	121,469
#2	(((((((((Congenital heart defect*[Text Word]) OR Congenital heart malformation*[Text Word]) OR CHD[Text Word]) OR Congenital heart disease*[Text Word]) OR Cardiac abnormalities[Text Word]) OR Cardiac abnormality[Text Word]) OR Cardiac defect*[Text Word]) OR Fetal heart[Text Word]) OR Fetal cardiac defect*[Text Word]) OR Foetal heart[Text Word]) OR Foetal cardiac defect*[Text Word]	105,996
#3	#1 OR #2<br>("Heart Defects, Congenital"[Majr]) OR ((((((((((Congenital heart defect*[Text Word]) OR Congenital heart malformation*[Text Word]) OR CHD[Text Word]) OR Congenital heart disease*[Text Word]) OR Cardiac abnormalities[Text Word]) OR Cardiac abnormality[Text Word]) OR Cardiac defect*[Text Word]) OR Fetal heart[Text Word]) OR Fetal cardiac defect*[Text Word]) OR Foetal heart[Text Word]) OR Foetal cardiac defect*[Text Word])	181,349
#4	(((("Body Mass Index"[Majr]) OR "Overweight"[Majr]) OR "Adiposity"[Majr]) OR "Thinness"[Majr]) OR "Metabolic Syndrome"[Majr]	185,465
#5	(((Maternal overweight[Text Word]) OR Maternal obesity[Text Word]) OR Prepregnancy BMI[Text Word]) OR Prepregnancy weight[Text Word]	3,677
#6	#4 OR #5<br>(((((("Body Mass Index"[Majr]) OR "Overweight"[Majr]) OR "Adiposity"[Majr]) OR "Thinness"[Majr]) OR "Metabolic Syndrome"[Majr])) OR ((((Maternal overweight[Text Word]) OR Maternal obesity[Text Word]) OR Prepregnancy BMI[Text Word]) OR Prepregnancy weight[Text Word])	187,082
#7	(("Pregnancy"[Majr]) OR "Infant"[Majr]) OR "Fetus"[Majr]	273,767
#8	(Offspring*[Text Word]) OR Pregnancy[Text Word]	975,184
#9	#7 OR #8<br>(((("Pregnancy"[Majr]) OR "Infant"[Majr]) OR "Fetus"[Majr])) OR ((Offspring*[Text Word]) OR Pregnancy[Text Word])	1,053,307
#10	#3 AND #6 AND #9<br>(((("Heart Defects, Congenital"[Majr]) OR ((((((((((Congenital heart defect*[Text Word]) OR Congenital heart malformation*[Text Word]) OR CHD[Text Word]) OR Congenital heart disease*[Text Word]) OR Cardiac abnormalities[Text Word]) OR Cardiac abnormality[Text Word]) OR Cardiac defect*[Text Word]) OR Fetal heart[Text Word]) OR Fetal cardiac defect*[Text Word]) OR Foetal heart[Text Word]) OR Foetal cardiac defect*[Text Word]))) AND (((((("Body Mass Index"[Majr]) OR "Overweight"[Majr]) OR "Adiposity"[Majr]) OR "Thinness"[Majr]) OR "Metabolic Syndrome"[Majr])) OR ((((Maternal overweight[Text Word]) OR Maternal obesity[Text Word]) OR Prepregnancy BMI[Text Word]) OR Prepregnancy weight[Text Word]))) AND ((((("Pregnancy"[Majr]) OR "Infant"[Majr]) OR "Fetus"[Majr])) OR ((Offspring*[Text Word]) OR Pregnancy[Text Word]))	114
#11	Updated search from 2019-10-07 to 2021-01-14<br>As #10	9

Embase Search Strategy

	Aspect 1	Aspect 2	Aspect 3
"MeSH" / Subject Heading<br><br>(Keyword + Map Term to Subject Heading + Explode + Include All Subheadings)	congenital heart malformation	exp body mass/ or exp obesity/ or exp underweight/<br><br>("Obesity" broader term for "metabolic syndrome X", "maternal obesity", "morbid obesity")	exp high risk pregnancy/ or exp pregnancy/ or infant/ or newborn/ or fetus/
Free text search<br><br>(Keyword)	Congenital heart defect* OR Congenital heart malformation* OR CHD OR Congenital heart disease* OR Cardiac abnormalities OR Cardiac abnormality OR Cardiac defect* OR Fetal heart OR Fetal cardiac defect* OR Foetal heart OR Foetal cardiac defect*	Maternal overweight OR Maternal obesity OR Prepregnancy BMI OR Prepregnancy weight	Offspring* OR Pregnancy

Embase Search 2019-10-06

		No. of articles
#1	exp congenital heart malformation/	126,712
#2	Congenital heart defect* OR Congenital heart malformation* OR CHD OR Congenital heart disease* OR Cardiac abnormalities OR Cardiac abnormality OR Cardiac defect* OR Fetal heart OR Fetal cardiac defect* OR Foetal heart OR Foetal cardiac defect*	128,917
#3	#1 OR #2	206,398
#4	exp high risk pregnancy/ or exp pregnancy/ or infant/ or newborn/ or fetus/	1,571,089
#5	Offspring* OR Pregnancy	882,813
#6	#4 OR #5	1,768,205
#7	exp body mass/ or exp obesity/ or exp underweight/	761,522
#8	Maternal overweight OR Maternal obesity OR Prepregnancy BMI OR Prepregnancy weight	7,737
#9	#7 OR #8	762,132
#10	#3 AND #6 AND #9	1,046
LIMITS	English Language + Exclude MEDLINE journals	91
#11	Updated search 2020-01-14	112

Maternal diabetes and CHDs in offspring

PubMed Search Strategy

	Aspect 1	Aspect 2	Aspect 3
MeSH	Heart defects, congenital	Diabetes Mellitus OR Pregnancy in diabetics OR Hyperglycemia OR Insulin Resistance	Pregnancy OR Infant OR Fetus
Free text search	(as above)	Pregestational diabetes OR PGDM OR Maternal diabetes OR Gestational diabetes OR GDM OR Diabetic women OR Diabetic pregnancies	(as above)

PubMed Search 2019-10-06

		No. of articles
#3	#1 OR #2 (as above)	181,349
#4	((("Diabetes Mellitus"[Majr]) OR "Pregnancy in Diabetics"[Mesh]) OR "Hyperglycemia"[Majr]) OR "Insulin Resistance"[Majr]	386,821
#5	((((((Pregestational diabetes[Text Word]) OR PGDM[Text Word]) OR Maternal diabetes[Text Word]) OR Gestational diabetes[Text Word]) OR GDM[Text Word]) OR Diabetic women[Text Word]) OR Diabetic pregnancies[Text Word]	20,405
#6	#4 OR #5	393,455

	((((("Diabetes Mellitus"[Majr]) OR "Pregnancy in Diabetics"[Mesh]) OR "Hyperglycemia"[Majr]) OR "Insulin Resistance"[Majr])) OR (((((((Pregestational diabetes[Text Word]) OR PGDM[Text Word]) OR Maternal diabetes[Text Word]) OR Gestational diabetes[Text Word]) OR GDM[Text Word]) OR Diabetic women[Text Word]) OR Diabetic pregnancies[Text Word])	
#9	#7 OR #8 (as above)	1,053,307
#10	#3 AND #6 AND #9<br>((((((("Diabetes Mellitus"[Majr]) OR "Pregnancy in Diabetics"[Mesh]) OR "Hyperglycemia"[Majr]) OR "Insulin Resistance"[Majr])) OR (((((((Pregestational diabetes[Text Word]) OR PGDM[Text Word]) OR Maternal diabetes[Text Word]) OR Gestational diabetes[Text Word]) OR GDM[Text Word]) OR Diabetic women[Text Word]) OR Diabetic pregnancies[Text Word]))) AND ((((("Pregnancy"[Majr]) OR "Infant"[Majr]) OR "Fetus"[Majr])) OR ((Offspring*[Text Word]) OR Pregnancy[Text Word]))) AND (("Heart Defects, Congenital"[Majr]) OR ((((((((((Congenital heart defect*[Text Word]) OR Congenital heart malformation*[Text Word]) OR CHD[Text Word]) OR Congenital heart disease*[Text Word]) OR Cardiac abnormalities[Text Word]) OR Cardiac abnormality[Text Word]) OR Cardiac defect*[Text Word]) OR Fetal heart[Text Word]) OR Fetal cardiac defect*[Text Word]) OR Foetal heart[Text Word]) OR Foetal cardiac defect*[Text Word]))	632
#11	Updated search from 2019-10-07 to 2021-01-14<br>As #10	43

Embase Search Strategy

	Aspect 1	Aspect 2	Aspect 3
”MeSH” / Subject Heading	congenital heart malformation	exp diabetes mellitus/ or exp hyperglycemia/ or exp insulin resistance/	exp high risk pregnancy/ or exp pregnancy/ or infant/ or newborn/ or fetus/
Free text search	(as above)	Pregestational diabetes OR PGDM OR Maternal diabetes OR Gestational diabetes OR GDM OR Diabetic women OR Diabetic pregnancies	(as above)

Embase Search 2019-10-06

		No. of articles
#3	#1 OR #2 (as above)	206,398
#6	#4 OR #5 (as above)	1,768,205
#7	exp diabetes mellitus/ or exp hyperglycemia/ or exp insulin resistance/	1,005,513
#8	Pregestational diabetes OR PGDM OR Maternal diabetes OR Gestational diabetes OR GDM OR Diabetic women OR Diabetic pregnancies	32,162
#9	#7 OR #8	1,008,162
#10	#3 AND #6 AND #9	2,001
LIMITS	English Language + Exclude MEDLINE journals	182
#11	Updated search 2020-01-14	217

Maternal hypertension or preeclampsia and CHDs in offspring

PubMed Search Strategy

	Aspect 1	Aspect 2	Aspect 3
MeSH	Heart defects, congenital	Hypertension OR Hypertension, pregnancy-induced	Pregnancy OR Infant OR Fetus
Free text search	(as above)	Maternal hypertension OR Maternal hypertensive disorder* OR Gestational hypertension	(as above)

PubMed Search 2019-10-06

		No. of articles
#3	#1 OR #2 (as above)	181,349
#4	("Hypertension"[Majr]) OR "Hypertension, Pregnancy-Induced"[Mesh]	211,800

#5	((Maternal hypertension[Text Word]) OR Maternal hypertensive disorder*[Text Word]) OR Gestational hypertension[Text Word]	3,466
#6	#4 OR #5<br>((("Hypertension"[Majr]) OR "Hypertension, Pregnancy-Induced"[Mesh])) OR (((Maternal hypertension[Text Word]) OR Maternal hypertensive disorder*[Text Word]) OR Gestational hypertension[Text Word])	213,166
#9	#7 OR #8 (as above)	1,053,307
#10	#3 AND #6 AND #9<br>((((("Hypertension"[Majr]) OR "Hypertension, Pregnancy-Induced"[Mesh])) OR (((Maternal hypertension[Text Word]) OR Maternal hypertensive disorder*[Text Word]) OR Gestational hypertension[Text Word])))) AND ((((("Pregnancy"[Majr]) OR "Infant"[Majr]) OR "Fetus"[Majr])) OR ((Offspring*[Text Word]) OR Pregnancy[Text Word]))) AND (("Heart Defects, Congenital"[Majr]) OR ((((((((((Congenital heart defect*[Text Word]) OR Congenital heart malformation*[Text Word]) OR CHD[Text Word]) OR Congenital heart disease*[Text Word]) OR Cardiac abnormalities[Text Word]) OR Cardiac abnormality[Text Word]) OR Cardiac defect*[Text Word]) OR Fetal heart[Text Word]) OR Fetal cardiac defect*[Text Word]) OR Foetal heart[Text Word]) OR Foetal cardiac defect*[Text Word]))	661
#11	Updated search from 2019-10-07 to 2021-01-14<br>As #10	25

Embase Search Strategy

	Aspect 1	Aspect 2	Aspect 3
”MeSH” / Subject Heading	congenital heart malformation	exp hypertension/ or exp "eclampsia and preeclampsia"/	exp high risk pregnancy/ or exp pregnancy/ or infant/ or newborn/ or fetus/
Free text search	(as above)	Maternal hypertension OR Maternal hypertensive disorder* OR Gestational hypertension	(as above)

Embase Search 2019-10-06

		No. of articles
#3	#1 OR #2 (as above)	206,398
#6	#4 OR #5 (as above)	1,768,205
#7	exp hypertension/ or exp "eclampsia and preeclampsia"/	735,885
#8	Maternal hypertension OR Maternal hypertensive disorder* OR Gestational hypertension	18,442
#9	#7 OR #8	736,285
#10	#3 AND #6 AND #9	2,637
LIMITS	English Language + Exclude MEDLINE journals	270
#11	Updated search 2020-01-14	311

Maternal dyslipidemia and CHDs in offspring

PubMed Search Strategy

	Aspect 1	Aspect 2	Aspect 3
MeSH	Heart defects, congenital	Dyslipidemias OR Lipoproteins, HDL	Pregnancy OR Infant OR Fetus
Free text search	(as above)	HDL OR Dyslipidemia*	(as above)

PubMed Search 2019-10-06

		No. of articles
#3	#1 OR #2 (as above)	181,349
#4	("Dyslipidemias"[Majr]) OR "Lipoproteins, HDL"[Majr]	66,228
#5	(Dyslipidemia*[Text Word]) OR HDL[Text Word]	103,854
#6	#4 OR #5<br>((("Dyslipidemias"[Majr]) OR "Lipoproteins, HDL"[Majr])) OR ((Dyslipidemia*[Text Word]) OR HDL[Text Word])	138,647
#9	#7 OR #8 (as above)	1,053,307
#10	#3 AND #6 AND #9<br>(#52 AND #96 AND #62)	87

	(((((("Dyslipidemias"[Majr]) OR "Lipoproteins, HDL"[Majr])) OR ((Dyslipidemia*[Text Word]) OR HDL[Text Word]))) AND ((((("Pregnancy"[Majr]) OR "Infant"[Majr]) OR "Fetus"[Majr])) OR ((Offspring*[Text Word]) OR Pregnancy[Text Word]))) AND (("Heart Defects, Congenital"[Majr]) OR ((((((((((Congenital heart defect*[Text Word]) OR Congenital heart malformation*[Text Word]) OR CHD[Text Word]) OR Congenital heart disease*[Text Word]) OR Cardiac abnormalities[Text Word]) OR Cardiac abnormality[Text Word]) OR Cardiac defect*[Text Word]) OR Fetal heart[Text Word]) OR Fetal cardiac defect*[Text Word]) OR Foetal heart[Text Word]) OR Foetal cardiac defect*[Text Word]))	
#11	Updated search from 2019-10-07 to 2021-01-14<br>As #10	0

Embase Search Strategy

	Aspect 1	Aspect 2	Aspect 3
”MeSH” / Subject Heading	congenital heart malformation	exp dyslipidemia/ or exp hypertriglyceridemia/ or exp high density lipoprotein/	exp high risk pregnancy/ or exp pregnancy/ or infant/ or newborn/ or fetus/
Free text search	(as above)	HDL OR Dyslipidemia*	(as above)

Embase Search 2019-10-06

		No. of articles
#3	#1 OR #2 (as above)	206,398
#6	#4 OR #5 (as above)	1,768,205
#7	exp dyslipidemia/ or exp hypertriglyceridemia/ or exp high density lipoprotein/	145,111
#8	HDL OR Dyslipidemia*	166,506
#9	#7 OR #8	202,390
#10	#3 AND #6 AND #9	187
LIMITS	English Language + Exclude MEDLINE journals	17
#11	Updated search 2020-01-14	17
