# Supplementary material for: Maternal obesity and metabolic disorders associate with congenital heart defects in the offspring: A systematic review
Source: PLoS One. 2021 May 27;16(5):e0252343. doi: 10.1371/journal.pone.0252343 (PMC8158948; doi:10.1371/journal.pone.0252343)
Supplement: S2 Table — (PDF) [file pone.0252343.s022.pdf]

Table S2: Newcastle-Ottawa Quality Assessment Scale – Case-control studies

Selection		
1. Is the case definition adequate?	A. yes, with independent validation	☆
	B. yes, eg record linkage or based on self-reports	
	C. no description	
2. Representativeness of the cases	A. consecutive or obviously representative series of cases	☆
	B. potential for selection biases or not stated	
3. Selection of Controls	A. community controls	☆
	B. hospital controls	
	C. no description	
4. Definition of Controls	A. no history of disease (endpoint)	☆
	B. no description of source	
Comparability		
1. Comparability of cases and controls on the basis of the design or analysis	A. study controls for ________________ (Select the most important factor.)	☆
	B. study controls for any additional factor (This criteria could be modified to indicate specific control for a second important factor.)	☆
Exposure		
1. Ascertainment of exposure	A. secure record (eg surgical records)	☆
	B. structured interview where blind to case/control status	☆
	C. interview not blinded to case/control status	
	D. written self-report or medical record only	
	E. no description	
2. Same method of ascertainment for cases and controls	A. yes	☆
	B. no	
3. Non-Response rate	A. same rate for both groups	☆
	B. non respondents described	
	C. rate different and no designation	

Note: A study can be awarded a maximum of one star for each numbered item within the Selection and Exposure categories. A maximum of two stars can be given for Comparability.

Further explanations and manuals for the scale can be found at:
http://www.ohri.ca/programs/clinical_epidemiology/oxford.asp

Table S2 (continued): Newcastle-Ottawa Quality Assessment Scale – Cohort studies

Selection		
1. Representativeness of the exposed cohort	A. truly representative of the average _______________ (describe) in the community	☆
	B. somewhat representative of the average ______________ in the community	☆
	C. selected group of users eg nurses, volunteers	
	D. no description of the derivation of the cohort	
2. Selection of the non-exposed cohort	A. drawn from the same community as the exposed cohort	☆
	B. drawn from a different source	
	C. no description of the derivation of the non-exposed cohort	
3. Ascertainment of exposure	A. secure record (eg surgical records)	☆
	B. structured interview	☆
	C. written self-report	
	D. no description	
4. Demonstration that outcome of interest was not present at start of study	A. yes	☆
	B. no	
Comparability		
1. Comparability of cohorts on the basis of the design or analysis	A. study controls for _______________ (Select the most important factor.)	☆
	B. study controls for any additional factor (This criteria could be modified to indicate specific control for a second important factor.)	☆
Outcome		
1. Assessment of outcome	A. independent blind assessment	☆
	B. record linkage	☆
	C. self-report	
	D. no description	
2. Was follow-up long enough for outcomes to occur	A. yes (select an adequate follow up period for outcome of interest)	☆
	B. no	
3. Adequacy of follow up of cohorts	A. complete follow up - all subjects accounted for	☆
	B. subjects lost to follow up unlikely to introduce bias - small number lost - > ____ % (select an adequate %) follow up, or description provided of those lost)	☆
	C. follow up rate < ____% (select an adequate %) and no description of those lost	
	D. no statement	

Note: A study can be awarded a maximum of one star for each numbered item within the Selection and Outcome categories. A maximum of two stars can be given for Comparability.

Further explanations and manuals for the scale can be found at:
http://www.ohri.ca/programs/clinical_epidemiology/oxford.asp
