# Supplementary material for: Maternal obesity and metabolic disorders associate with congenital heart defects in the offspring: A systematic review
Source: PLoS One. 2021 May 27;16(5):e0252343. doi: 10.1371/journal.pone.0252343 (PMC8158948; doi:10.1371/journal.pone.0252343)
Supplement: S3 Table — Studies listed after number of CHD cases: Studies with most cases first etc. Abbreviations: ABDCCS, Atlanta Birth Defect Case-Control Study; BDRFSS, Birth Defects Risk Factor Surveillance Study; CHDs, congenital heart defects; CM, congenital malformations; CSL, The Consortium of Safe Labor; DM1, diabetes mellitus type 1; DM2, diabetes mellitus type 2; GDM, gestational diabetes mellitus; NBDPS, National Birth Defects Prevention Study; NOS, Newcastle Ottawa Scale; PE, preeclampsia; PGDM, pregestational diabetes. (PDF) [file pone.0252343.s023.pdf]

Table S3: Characteristics of included studies

Author/publication year	Country of study population	Study design	Population size	Cases with CHDs	Included study population	Plurality	Maternal exposure	Sources of maternal exposure	NOS score
Hoang 2016 [17]	USA (Texas)	Cohort	4 207 898	48 249	Live births	All	PGDM (not specified), GDM	Registers	8
Persson 2019 [14]	Sweden	Cohort	2 050 491	28 628	Live births	Singletons	Obesity	Registers	8
Liu 2013 [19]	Canada (excl. Quebec)	Cohort	2 278 838	26 488	Live births	All	Obesity, DM1, DM2, hypertension	Registers	8
Chou 2016 [48]	Taiwan	Cohort	1 387 650	23 483	Live births	All	DM1, DM2, hypertension	Registers	8
Boyd 2017 [21]	Denmark	Cohort	1 972 857	18 038	Live births	Singletons	PE	Registers	8
Auger 2015 [22]	Canada (Quebec)	Cohort	1 942 072	17 296	Live births	All	PE	Registers	8
Ludvigsson 2018 [13]	Sweden	Cohort	1 162 323	17 242	Live births	Singletons	DM1	Registers	8
Øyen 2016 [16]	Denmark	Cohort	2 025 727	16 325	Live births	Singletons	PGDM (DM1 or DM2), GDM	Registers	8
Blomberg 2010 [30]	Sweden	Cohort	1 049 582	11 163	Live births, stillbirths	Not stated	Obesity	Registers	8
Fisher 2017 [20]	USA	Case-control (NBDPS)	21 762	10 625	Live births, stillbirths, terminated	Singletons	Untreated hypertension	Interviews	8
Leirgul 2016 [18]	Norway	Cohort	914 427	10 575	Live births, stillbirths, terminated	Singletons	PGDM (DM1, DM2 or unspecified DM), GDM	Registers	8
Block 2013 [29]	USA (Florida)	Case-control	1 124 370	9 314	Live births	Singletons	Obesity	Registers	7
Mills 2010 [32]	USA (New York State)	Case-control (nested)	63 696	7 392	Live births	Singletons	Obesity	Medical records	7
Cedergren 2003 [35]	Sweden	Case-control	812 457	6 801	Live births, stillbirths	Not stated	Obesity	Registers	7
Gilboa 2010 [31]	USA	Case-control (NBDPS)	12 113	6 440	Live births, stillbirths	Not stated	Obesity, obesity+GDM	Interviews	8
Correa 2008 [46]	USA	Case-control (NBDPS)	17 925	4 621	Live births	Not stated	PGDM (DM1 or DM2), GDM	Interviews	8
Vereczkey 2014 [43]	Hungary	Case-control	41 713	3 562	Live births	All	PGDM (DM1 or DM2), hypertension	Registers	8
Brodwall 2016 [50]	Norway	Cohort	914 703	2 473	Live births, stillbirths, terminated	Singletons	PE	Registers	8

Sharpe 2005 [49]	Australia (South Australia)	Cohort	282 260	2 418	Live births, stillbirths	Singletons	DM1, GDM (GDM or impaired glucose tolerance)	Interviews	8
Liu 2015 [39]	China (Tianjin)	Cohort	90 796	1 817	Live births	All	Obesity, GDM	Questionnaire / medical records	8
Brite 2014 [15]	USA	Cohort (CSL)	121 815	1 388	Live births	Singletons	Obesity	Medical records	8
Yuan 2020 [42]	China	Case-control	2 318	1 206	Live births, stillbirths, terminated	Not stated	Overweight	Medical records	8
Watkins 2001 [37]	USA	Case-control (ABDCCS)	4 078	1 049	Live births, stillbirths	Not stated	Obesity	Interviews	7
Agopian 2012 [40]	USA (Texas)	Cohort	3 806 299	563	Live births, stillbirths, terminated	All	Obesity, PGDM (not specified), GDM	Registers	8
Rankin 2010 [33]	England	Cohort	41 013	341	Live births, stillbirths, terminated	Singletons	Obesity	Registers	8
Shaw 2008 [34]	USA (California)	Case-control	1 359	323	Live births, stillbirths, terminated	Not stated	Obesity	Interviews	8
Dolk 2020 [41]	Northern Ireland	Case-control	1 208	242	Live births, stillbirths	Not stated	Obesity, PGDM (not specified), GDM	Questionnaire / medical records	8
Kovalenko 2018 [44]	Russia (Murmansk)	Cohort	52 253	233	Live births	Singletons	PGDM (DM1 or DM2)	Registers	8
Shaw 2000 [38]	USA (California)	Case-control	2 033	202	Live births, stillbirths	Not stated	Obesity	Interviews	7
Watkins 2003 [36]	USA	Case-control (Atlanta BDRFSS)	525	195	Live births, stillbirths, terminated	Not stated	Obesity	Interviews	7
Vinceti 2014 [45]	Italy	Case-control (nested)	479 720	68	Live births, stillbirths	All	PGDM (DM1 or DM2)	Registers	8
Erickson 1991 [47]	USA	Case-control (ABDCCS)	7 900	35	Live births, stillbirths	Not stated	PGDM (not specified)	Interviews	7

Note: Studies listed after number of CHD cases: studies with most cases first etc.

Abbreviations: ABDCCS, Atlanta Birth Defect Case-Control Study; BDRFSS, Birth Defects Risk Factor Surveillance Study; CHDs, congenital heart defects; CM, congenital malformations; CSL, The Consortium of Safe Labor; DM1, diabetes mellitus type 1; DM2, diabetes mellitus type 2; GDM, gestational diabetes mellitus; NBDPS, National Birth Defects Prevention Study; NOS, Newcastle Ottawa Scale; PE, preeclampsia; PGDM, pre-gestational diabetes.
