# Supplementary material for: Maternal obesity and metabolic disorders associate with congenital heart defects in the offspring: A systematic review
Source: PLoS One. 2021 May 27;16(5):e0252343. doi: 10.1371/journal.pone.0252343 (PMC8158948; doi:10.1371/journal.pone.0252343)
Supplement: S4 Table — The Newcastle-Ottawa Quality Assessment Scale is explained in detail in S2 Table. (PDF) [file pone.0252343.s024.pdf]

Table S4: Newcastle-Ottawa Quality Assessment Scale (NOS) score for each study included in the review

Author/publication year	Study design	Individual components of the NOS			Comments
		Selection (4 p)	Comparability (2 p)	Exposure/outcome (3 p)	
Hoang 2016 [17]	Cohort	3	2	3	Maternal exposure from registers (-1 p)
Persson 2019 [14]	Cohort	3	2	3	Maternal exposure from registers (-1 p)
Liu 2013 [19]	Cohort	3	2	3	Maternal exposure from registers (-1 p)
Chou 2016 [48]	Cohort	3	2	3	Maternal exposure from registers (-1 p)
Boyd 2017 [21]	Cohort	3	2	3	Maternal exposure from registers (-1 p)
Auger 2015 [22]	Cohort	3	2	3	Maternal exposure from registers (-1 p)
Ludvigsson 2018 [13]	Cohort	3	2	3	Maternal exposure from registers (-1 p)
Øyen 2016 [16]	Cohort	3	2	3	Maternal exposure from registers (-1 p)
Blomberg 2010 [30]	Cohort	3	2	3	Maternal exposure from registers (-1 p)
Fisher 2017 [20]	Case-control	4	2	2	No information about interview being blinded (-1 p)
Leirgul 2016 [18]	Cohort	3	2	3	Maternal exposure from registers (-1 p)
Block 2013 [29]	Case-control	3	3	2	Cases defined from record linkage (-1 p); Maternal exposure from medical records (-1 p)
Mills 2010 [32]	Case-control	3	3	2	Cases defined from record linkage (-1 p); Maternal exposure from medical records (-1 p)
Cedergren 2003 [35]	Case-control	3	3	2	Cases defined from record linkage (-1 p); Maternal exposure from medical records (-1 p)
Gilboa 2010 [31]	Case-control	4	2	2	No information about interview being blinded (-1 p)
Correa 2008 [46]	Case-control	4	2	2	No information about interview being blinded (-1 p)
Vereczkey 2014 [43]	Case-control	4	2	2	Maternal exposure from medical records (-1 p)
Brodwall 2016 [50]	Cohort	3	2	3	Maternal exposure from registers (-1 p)
Sharpe 2005 [49]	Cohort	3	2	3	Maternal exposure from registers (-1 p)
Liu 2015 [39]	Cohort	3	2	3	Maternal exposure from questionnaires (-1 p)
Brite 2014 [15]	Cohort	3	2	3	Maternal exposure from medical records (-1 p)
Yuan 2020 [42]	Case-control	4	2	2	Maternal exposures from non-blinded interviews or/and from medical records (-1 p)
Watkins 2001 [37]	Case-control	3	2	2	Cases defined from record linkage (-1 p); No information about interview being blinded (-1 p)
Agopian 2012 [40]	Cohort	3	2	3	Maternal exposure from registers (-1 p)
Rankin 2010 [33]	Cohort	3	2	3	Maternal exposure from registers (-1 p)
Shaw 2008 [34]	Case-control	4	2	2	Exposure from non-blinded interviews (-1 p)
Dolk 2020 [41]	Case-control	4	2	2	Maternal exposures were self-reported or/and from medical records (-1 p)
Kovalenko 2018 [44]	Cohort	3	2	3	Maternal exposure from registers (-1 p)
Shaw 2000 [38]	Case-control	4	1	2	Not adjusted for maternal age (-1 p); Exposure from non-blinded interviews (-1 p)
Watkins 2003 [36]	Case-control	3	2	2	Cases defined from record linkage (-1 p); No information about interview being blinded (-1 p)
Vinceti 2014 [45]	Case-control	3	2	3	Maternal exposure from registers (-1 p)
Erickson 1991 [47]	Case-control	3	2	2	Cases defined from record linkage (-1 p); Maternal exposure from partly blinded interviewers (-1 p)

Note: The Newcastle-Ottawa Quality Assessment Scale is explained in details in S2 Table.
